# The ϕ6 Cystovirus Protein P7 Becomes Accessible to Antibodies in the Transcribing Nucleocapsid: A Probe for Viral Structural Elements

**DOI:** 10.1371/journal.pone.0122160

**Published:** 2015-03-23

**Authors:** Alexandra Alimova, Hui Wei, Al Katz, Linda Spatz, Paul Gottlieb

**Affiliations:** 1 Sophie Davis School of Biomedical Education, City College of New York, New York, NY 10031, United States of America; 2 Department of Physics, City College of New York, New York, NY 10031, United States of America; New York State Dept. Health, UNITED STATES

## Abstract

Protein P7 is a component of the cystovirus viral polymerase complex. In the unpackaged procapsid, the protein is situated in close proximity to the viral directed RNA polymerase, P2. Cryo-electron microscopy difference maps from the species ϕ6 procapsid have demonstrated that P7 and P2 likely interact prior to viral RNA packaging. The location of P7 in the post-packaged nucleocapsid (NC) remains unknown. P7 may translocate closer to the five-fold axis of a filled procapsid but this has not been directly visualized. We propose that monoclonal antibodies (Mabs) can be selected that serve as probe- reagents for viral assembly and structure. A set of Mabs have been isolated that recognize and bind to the ϕ6 P7. The antibody set contains five unique Mabs, four of which recognize a linear epitope and one which recognizes a conformational epitope. The four unique Mabs that recognize a linear epitope display restricted utilization of V_κ_ and V_H_ genes. The restricted genetic range among 4 of the 5 antibodies implies that the antibody repertoire is limited. The limitation could be the consequence of a paucity of exposed antigenic sites on the ϕ6 P7 surface. It is further demonstrated that within ϕ6 nucleocapsids that are primed for early-phase transcription, P7 is partially accessible to the Mabs, indicating that the nucleocapsid shell (protein P8) has undergone partial disassembly exposing the protein’s antigenic sites.

## Introduction

The cystoviridae family of viruses, of which ϕ6 was the first discovered species, contain three segments of double stranded RNA. Bacteriophage ϕ6 and its relatives are model systems for virus assembly, genome packaging and dsRNA polymerization. The RNA packaging, replication, transcription mechanism, and overall structure resembles that of reoviruses making the species an excellent model system to study these important pathogens. The initial step in cystoviridae replication is the assembly of a closed and unexpanded, dodecahedral-shaped procapsid (PC). The RNA packaging proceeds in a specific order with the small (2948 bp) viral RNA segment packaged first, followed by the middle (4063 bp) and large (6374 bp) segments [1–3]. Step-wise expansion of the PC accompanies the RNA packaging [4]. Ultimately all three ds-RNA segments are enclosed into a nucleocapsid (NC) surrounded by a lipoprotein envelope to constitute the mature viral particle. The outer layer of the NC is a shell composed of a matrix assembled of protein P8 [5–7] that upon cell penetration facilitates an endocytic plasma membrane penetration and is thought to disassemble during viral entry [8]. The P8 shell is composed of 200 trimers arranged as a T = 13 lattice that partially covers the filled PC [5,9,10].

During genome packaging the PC undergoes significant conformational morphogenesis with the sequential expansion revealing unique binding sites for each of the three viral RNA segments [11,12]. The PC is composed of four proteins, P1, P2, P4, and P7, which are responsible for RNA packaging, transcription, and genome replication [11,13]. Three of the four proteins (P1, P2 and P4) are known to have specific functions in regard to the packaging and replication of viral RNA. The entire PC framework is composed of P1 which has RNA binding activity. The atomic structure of P1 for both ϕ6 and ϕ8 has recently been determined and shown to be a flattened trapezoid in shape that adapts to two conformations, P1A and P1B, that undergo conformational changes when maturing from the unexpanded PC to the RNA packaged NC [14–16]. A hexamer of the nucleotide triphosphorylase, P4, forms the packaging portal responsible for RNA transport into the expanding PC. The viral RNA-directed RNA polymerase (RdRP), P2, is required for the replication of the single stranded RNA to the double-stranded RNA (dsRNA) genome [5].

P7 is the least characterized of the PC proteins and its precise function still remains undetermined. It is required for efficient PC assembly and transcription [17], and RNA packaging [18,19]. In ϕ6, P7 has a molecular mass of 17168 Da. The ϕ6 virion can potentially contain 60 copies of P7 (three copies at each of the 20 three-fold symmetry axes); but there is a controversy regarding occupancy in recombinant PC particles: SunBamford and Poranen [20] noted that the same amount of P7 is in recombinant PC particles as in the complete virion. Our previous publication described approximately 20 copies of P7 protein per PC particle [21]; while NemecekQiaoMindichSteven and Heymann [16] observed even less occupancy for P7, at only 12 copies in a complete PC. The occupancy of P7 in mature viruses has not been determined and may differ from recombinant PC particles. There is evidence that P7 forms an elongated dimer in solution [17], but in both the PC and NC, P7 is seen to exist as a monomer. PoranenButcherSimonovLaurinmaki and Bamford [22] observed that an excess concentration of P7 accelerated assembly of P1 *in vitro*, indicating that P7 may stabilize P1. Study of self-assembly of PC particles shows potential competition between P2 and P7 proteins [16]. In the presence of an excess of P7, the number of P2 copies decreases from 12 to 6 copies, whereas the number of P7 copies increases from 36 to 60 copies per particle [20]. In prior work, we proposed that P7 is located “inside” the unexpanded PC core near the three-fold axes and that P7 stabilizes P2 in position close to the three-fold axis [21].

The structure of P7 from the related cystovirus ϕ12 has been determined by X-ray crystallography [23]. Most interestingly, the protein is seen to be composed of two distinct domains in which the N-terminal core region (1–129) of P7 forms a homodimeric α/β-fold, having structural similarities with breast cancer gene 1 (BRCA1) C-terminal domains implicated in multiple protein–protein interactions in DNA repair proteins. The C-terminal tail consisting of approximately 30 to 40 amino acids is less ordered structurally and nuclear magnetic resonance analysis strongly suggests that it is capable of interacting with the viral RNA and the RdRP. Recently, difference maps generated from single particle reconstructions show that in ϕ6, P7 is located very near the inner three-fold axis of symmetry in the unpackaged and unexpanded PC [21]. In that work, it was noted that P7 interacts with P2, and it is proposed that the protein stabilizes the location of P2 near the inner three-fold axis.

Juuti and Bamford [17] noted that P7 is accessible to polyclonal antibodies from the NC surface and that protein P8 is released by the polyclonal antibodies. While the NC structure is well-established [5,10], actively transcribing NCs have not been directly observed and the precise position and translocation of the RNA portal protein elements have not been determined. In this paper, we report the isolation of a set of monoclonal antibodies (Mabs) with specificity to unique epitopes of P7. These Mabs are utilized as reagents for a first determination of P7 accessibility in a transcribing NC structure. We note that under conditions that favor early-phase ϕ6 transcription, P7 is accessible to antibodies *in situ*. The Mabs show potential as reagents to reveal viral structure during assembly and disassembly.

## Materials and Methods

### Cloning, expression, and purification of P7

The ϕ6 P7 coding region was polymerase chain reaction (PCR) amplified from a pLM687 plasmid template [24] using oligonucleotides OL161 5’-TACTGACAATCCTGTCGTTA-3’ and OL162 5’-TCATCGACATCGAACGGTTG-3’ as upstream and downstream primers, respectively. The PCR product was initially inserted into PCR2.1 TOPO and the recombinant plasmid, designated pPG126, was double digested with restriction endonucleases Ndel and EcoRI. A 498 bp fragment was isolated and ligated with pET28-a vector (Novagen, Madison, WI) at the Ndel and EcoRI sites. The ligation product was transformed in *E*. *coli* XL-1 blue supercompetent cells (Novagen, Madison, WI) and selected for kanamycin resistance (50 μg/ml). The positive clone selected was designated pPG128 and was subsequently transformed into T7 Express *lysY/Iq* Competent *E*. *coli* (New England Biolabs, Ipswich, MA). Five ml of overnight culture of the transformants were inoculated in 250 ml Luria-Bertani liquid media, supplemented with 50 μg/ml of kanamycin, and grown at 27°C for 14 h. P7 protein expression was then induced by adding 1 mM of isopropyl-thio-galactoside (IPTG) and the cells were grown at 27°C for an additional 4 h. The expressed protein was visualized by using 12% SDS-PAGE gel of the cell’s samples before and after induction. Induced cells were pelleted by centrifugation at 7000g for 30 min at 4°C and the pellets were stored at −80°C.

The frozen pellets were thawed on ice and re-suspended in 5 ml of lysis buffer consisting of 50 mM of NaH_2_PO_4_, 500 mM of NaCl, 1 mM of MgCl_2_, 10 mM of imidazole and protease inhibitor cocktail (Roche, Nutley, NJ). The cells were sonicated and lysed on ice by using a Microtip Misonix S-4000 sonicator (Qsonica LLC Newtown, CT), at 50% power (13 W) for 10 min, with a repeating power cycle of 10 s ON, 10 s OFF. In order to remove cells debris, the sonicated samples were centrifuged at 20000g for 30 min. The supernatant containing soluble P7 was mixed with Ni-NTA His-Bind resin (Novagen, Madison, WI) pre-equilibrated with washing buffer (50 mM of NaH_2_PO_4_, 540 mM of NaCl, 1 mM of MgCl_2_, 1% glycerol, 20 mM of Imidazole, pH 8.0) and incubated for 2 h in a shaker at room temperature. A gravity flow column was packed with beads incubated with the cell’s supernatant and washed with 50 ml of washing buffer. P7 was eluted with 3 ml of elution buffer (50 mM of NaH_2_PO_4_, 540 mM of NaCl, 1 mM of MgCl_2_, 1% glycerol, 250 mM of imidazol, pH 8.0) and the sample was desalted and concentrated by using Amicron (Millipore, Billerica, MA) 10 kDa cutoff filter.

The P7 concentration was estimated from the density of the band on a Coomassie-stained SDS-PAGE gel. The density data were compared with known amounts of lysozyme protein (MW 14.3 kDa) loaded on the same gel. The amount of protein was estimated by integrating the area under the band curve and applying a background correction.

### NC purification

The growth and purification of ϕ6 followed the procedure described by Mindich et al. [25]. The viral envelope was removed with 1% Triton X-100 to yield isolated NCs by the procedure similar to the one outlined by de HaasPaateroMindichBamford and Fuller [26]. Analysis of virion size (NC diameter is ~58 nm; complete virion diameter is ~86 nm) in TEM micrographs indicated that this procedure resulted in near total removal of the viral envelope.

### Antibody production

Four BALB/c female mice were each immunized with 50 μg of P7 in complete Freund’s adjuvant (CFA) on day 0. The mice were boosted on days 21 and 51 with 25 μg and 50 μg of native P7 in incomplete Freund’s adjuvant (IFA) respectively. The mice were bled immediately before each immunization and the sera samples were tested for anti-P7 antibodies by ELISA. One week after the first immunization, the mice were bled and the sera samples were tested for the presence of anti-P7 IgM antibody. The mice were then bled four days after the 1^st^ boost and tested for the presence of anti-P7 IgG antibody. The second boost was performed four days before fusion. Spleen cells from immunized mice were fused with NSO myeloma cell (Sigma Aldrich, St. Louis, MO) using polyethylene glycol 4000 and the hybridoma cells were grown in hypoxanthine aminopteran thymidine (HAT) selection medium.

Hybridomas secreting antibodies that were positive by ELISA for P7 antigen were subcloned by limiting dilution in complete HAT-DME (Dulbecco’s Modified Eagles) medium supplemented with 10% fetal bovine serum, 1% glutamine, 10% NCTC-109 (Life Technologies, Grand Island, NY), 1% of MEM nonessential amino acids (Corning cellgro, Manassas, VA), and 1% penicillin-streptomycin. Hybridoma Cloning Factor (Thermo Fisher Scientific, Waltham, MA) was used to support cell growth. Only subclones, whose secreted antibodies bound P7 but were non-reactive to BSA and a 6-histidine tagged EBNA-1 protein were selected for future propagation. The His-tag EBNA protein was used as a negative control for 6x-his antigen. Initially five selected subcloned hybridomas were grown in Serum-Free DME medium (Sigma-Aldrich, St Louis, MO) to obtain a total of 500 ml of supernatants. All the supernatants were tested by ELISA for anti-P7 IgG antibody and the Mabs labeled as 6C3, 2D11, 1F11, 1B6 and 1C10. A germline analysis search demonstrates that 2D11 is similar to 1B6; 1C10 is similar to 1F11; and therefore, could be derived from the same myeloma fusion (Table 1). In Table 1, the germline gene with the highest alignment is recorded.

**Table 1:** Germline Assignments (IgBLAST).

Mab	V_H_	J_H_	# mutations	V_κ_	J_κ_	# mutations
1B6	V5-9-3*01	2	18	V4-59*01	5	9
2D11	V5-9-3*01	2	20	V4-59*01	5	10
1C10	V5-17*02	4	5	V4-59*01	5	5
1F11	V5-17*02	4	5	V4-59*01	5	9
6C3	V2-9-1*01	3	7	V8-21*01	4	11

Anti-P7 IgG Mabs were purified from the supernatants by using Gamma Bind Plus Sepharose (GE Healthcare Life Sciences, Pittsburgh, PA). The antibody was eluted with 8 ml of 0.1 M glycine at pH 3.0 and immediately adjusted to neutral pH with 800 μl of 1 M Tris-HCl at pH 8.0. The antibody was de-salted and concentrated by an Amicron 10 kDa cut-off filter. Antibody concentration was determined from optical density with a NanoDrop spectrophotometer (NanoDrop division of ThermoScientific, Wilmington, DE).

### Animal Welfare

The IACUC of The City College of New York/CUNY Medical School approved this study—Approval Number 832.2. Mice were anesthetized with isofluorane prior to blood drawing and the mice are euthanized by deep isofluorane inhalation followed by cervical dislocation or CO_2_ asphyxiation.

### Isotype ELISAs

The Mabs were isotyped by using an indirect ELISA method. Serial dilutions of the Mabs, starting from an initial concentration of 500 ng/ml were added to wells, and plates were incubated for 1 h at 37°C. The isotype specificity of the Mabs was detected by incubating coated plates with the following goat anti-mouse antibodies conjugated to alkaline phosphatase: anti-IgG1, anti-IgG2a, anti-IgG2b, anti-IgG3 anti-kappa and anti-lambda for 1 h at 37°C. Phosphatase Substrate (Sigma-Aldrich) was added and color development was measured using a Titertek ELISA reader at 405 nm.

### cDNA synthesis

Total RNA from 100% confluent T25 flasks were extracted for all four hybridoma cultures by using the TRIzol method, iso-propanol concentrated, and air-dried. The RNA was resuspended in 50 μl of Diethylpyrocarbonate (DEPC) water. The first strand of cDNA was synthesized from the RNA template by using the SuperScript III System for RT-PCR (Invitrogen, Thermo Fisher Scientific, Waltham, MA). Briefly, the 2 μl of total RNA isolated from T25 flasks was reverse transcribed in a reaction volume of 20 μl containing 1 mM of oligo(dT)_20_, 10 nmol of each of four dNTPs, 40 U of RNaseOUT, 1x RT buffer, 100 nmol of MgCl_2_, 100 U of SuperScript III reverse transcriptase and 0.2 nmol of dithiothreitol (DTT). Reverse transcription reactions were performed at 50°C for 50 min followed by 85°C for 5 min. Then 1 μl of RNase H was added and samples were incubated at 37°C for 20 min.

Immunoglobulin V_H_ and V_κ_ transcripts were amplified independently by PCR (High-Fidelity Phusion PCR kit by Finnzymes, Fisher Scientific, Pittsburgh, PA) using 2 μl of RT reaction product as a template. Universal V_H_ forward and reverse γ1 primers and universal forward V_κ_ and reverse κ primers. were designed by TillerBusse and Wardemann [27]. All PCRs were performed in 50 μl total reaction volume at 94°C for 15 s followed by 50 cycles at 94°C for 30 s; 60°C (V_H_) or 50°C (V_κ_) for 30 s; 72°C for 45 s (V_H_) or 55 s (V_κ_); and a final extension at 72°C for 10 min. Amplified PCR products were gel purified by using QIAquick Gel Extraction Kit (Qiagen, Valencia, CA). Purified products were sequenced (Macrogen Service, Rockville, MD).

### Western Blot

For the western blot analysis, 0.3 μg of purified P7 was loaded on to a 12% SDS-PAGE gel. Identical loadings were performed for each of the five Mabs. After gel separation, the proteins were transferred onto a nitrocellulose transfer membrane (BA-S 85 by OPTITRAN) and stained with 0.1% Ponceau S (Sigma-Aldrich, St. Louis, MO) in 5% glacial acetic acid to visualize the transferred bands. The membranes were then cut into five sections for each of the Mabs. The membranes were blocked in 5% skim milk and 0.01% Tween20 in PBS, and then incubated overnight in the presence of 1 μg/ml of each Mab in PBS and 0.01% Tween20. The membranes were then incubated in the presence of a 1:20000 dilution of goat anti-mouse IgG conjugated with HRP (horse radish peroxidase) added to 3% skim milk and 0.01% Tween20 in PBS. To develop the Western Blot Marker (Magic Mark XP Western Standard by Invitrogen) the 1 μl of Precision Protein StrepTactin-HRP conjugated (Bio-Rad Life Sciences, Hercules, CA) was added to each of 10 ml of secondary antibody solution. The membranes were incubated in Pierce ECL Western Blotting Substrate (Thermo Fisher Scientific, Rockford, IL) and then Kodak X-OMAT LS film was exposed to visualize the HRP luminescence.

### ELISAs to determine whether two Mabs compete for the same or different epitopes on P7

To determine whether two Mabs bound to the same or different epitopes on P7, a mixture of two anti-P7 Mabs were applied to P7 coated ELISA plates. The ELISA plates were coated with 25 ng/ml (1.25 ng per well of native P7). The optimal concentration of P7 antigen per well was obtained by titrating P7 to determine the linear range of activity. One of the Mabs was kept at a constant concentration of 1000 ng/ml (saturation concentration) while the concentration of the other competing Mab was varied in the range of 3 ng/ml to 1000 ng/ml. As a control, a non-competing ELISA was performed for each of the five Mabs in a range of concentrations from 3 ng/ml to 1000 ng/ml. Secondary anti-mouse IgG antibodies conjugated to alkaline phosphatase were added followed by substrate solution.

### 6C3 Mab biotinylation

The 6C3 Mab was biotinylated by the ChromaLink Biotin One-shot antibody labeling kit, product of Solulink Inc. (San Diego, CA, USA). The biotinylation process was monitored by measuring changes in the optical absorption of the reaction mixture. The calculated biotin molar substitution ratio is 8.35, indicating efficient binding of 6C3. A total of 86 μg of antibody was recovered after the reaction.

### Transcription reaction

The transcription reaction was initiated by incubating 200 μg of NC in 200 μl of transcription reaction mixture [pH 8.8; 70 mM of bicine; 1 mM of Mg Acetate; 3 mM of MnCl_2_; 125 mM of NH_4_Cl; 20 mM of DTT; 0.25 U/L of RNasin; approximately 100 μg of macaloid (bentonite clay); and 4 mM of each of the rNTPs] for 1 h at 20°C [28]. As a control, the same amount of NC was utilized in the transcription reaction mixture but without the addition of rNTPs. For each sample, 45 μL was used for RNA transcript extraction. Samples were electrophoresed in 1% agarose gels and stained with ethidium bromide. The cryo-TEM visualization of transcribing NCs used 5 μl each of the control and reaction samples. Electron micrographs were acquired with a JEOL 2100 electron microscope (JEOL Inc. Peabody, MA, USA) operating at 200 keV and a Gatan Orius 2048 **×** 2048 pixel CCD (Gatan Inc., Pleasanton, CA, USA).

### Sandwich ELISA of transcribing NCs

Two Mabs with different epitope recognition, 1C10 and 6C3, were chosen for the sandwich-based ELISA of the NC. The capture antibody was 1C10 and the detecting antibody was biotinylated 6C3. ELISA plates were coated with 5 μg/ml of 1C10 (the Mabs were dissolved in 0.2 M of carbonate-bicarbonate buffer at pH 9.4). The plates were then blocked with 1% BSA in PBS. The transcription reaction mixture (50 μl) was applied to the first well and subsequent 2-fold dilutions with PBS were applied to the remaining wells. Two control samples consisted of: (1) NCs in 100 mM Tris buffer and (2) NCs in transcription buffer but lacking rNTPs were employed for comparison. Following the addition of biotinylated 6C3, plates were incubated with a 1:1000 dilution of streptavidin conjugated with alkaline phosphatase. Substrate solution was added and plates were read at 405 nm using a Titertek Multiscan ELISA reader (TiterTek, Berthold, Pforzheim, Germany).

## Results and Discussion

### Isolation of ϕ6 P7, inoculation of mice, and genetic classification of the monoclonal antibodies

**Fig 1:**
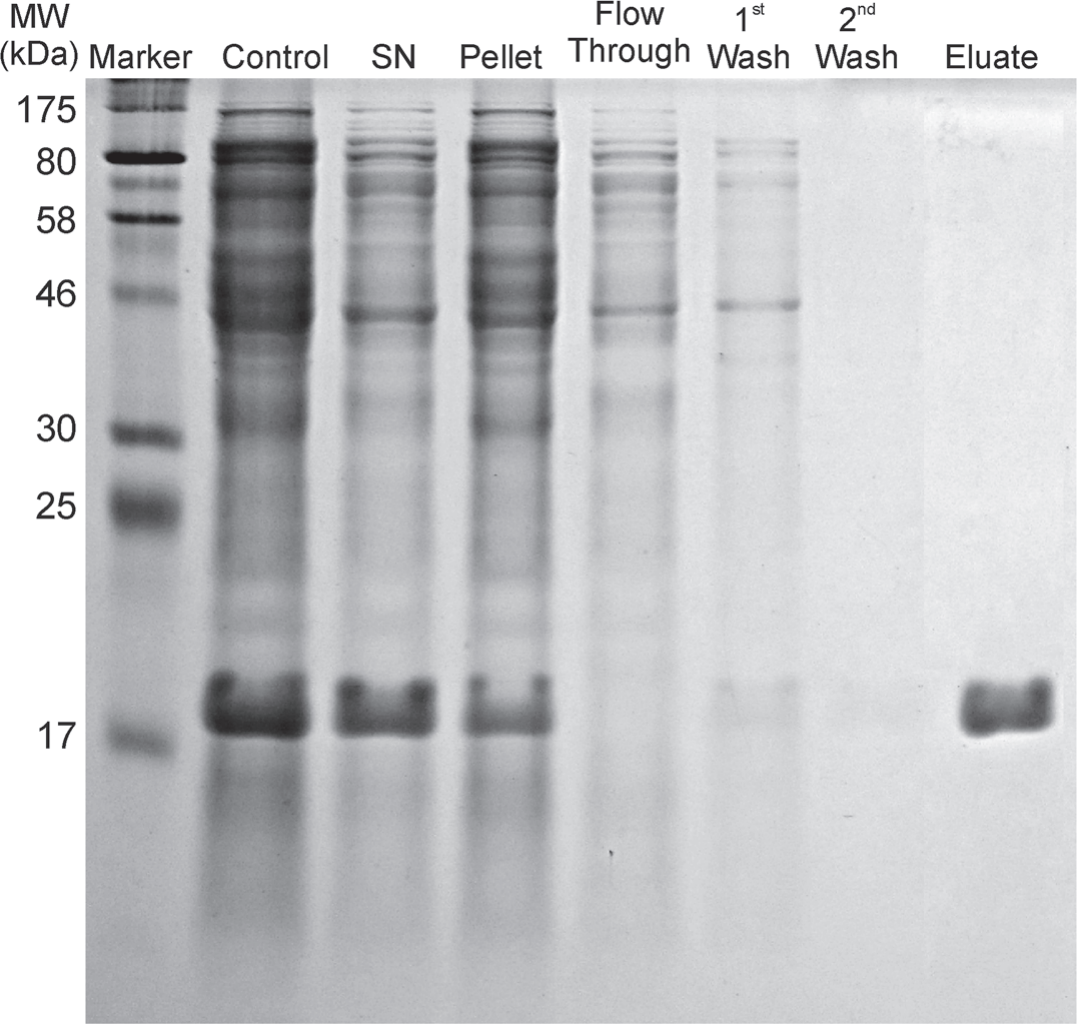
SDS-PAGE gel demonstrating effectiveness of P7 purification procedure. Lanes are: Marker (wide range protein ladder p7708); Control—undisrupted cells after IPTG induction; SN—supernatant after the pelleting of lysed cell’s debris; Pellet—resuspended cell debris pellet; Flow-Through—proteins unbound to Ni-NTA beads; 1^st^ and 2^nd^ Wash—proteins eluted from the Ni-NTA agarose column after 1^st^ and 2^nd^ rinses with washing buffer; Eluate—P7 protein eluted from the column.

The SDS-PAGE gel taken at different steps of the P7 purification procedure is shown in Fig. 1 and demonstrates that recombinant his-tagged protein P7 is produced to a high percentage in *E*. *coli* in a soluble state. The protein was bound to Ni-NTA beads efficiently eluted with 250 mM of imidazole. Purity was estimated from the coomassie-stained SDS-PAGE and the P7 protein was observed to migrate at a molecular mass of approximately 17 kDa. 50 μg of native form P7 in CFA was used to intraperitoneally inoculate 6-week-old female, BALB/c mice. Two booster inoculations which included P7 emulsified inIFA were administered at days 21 and 51.

**Fig 2:**
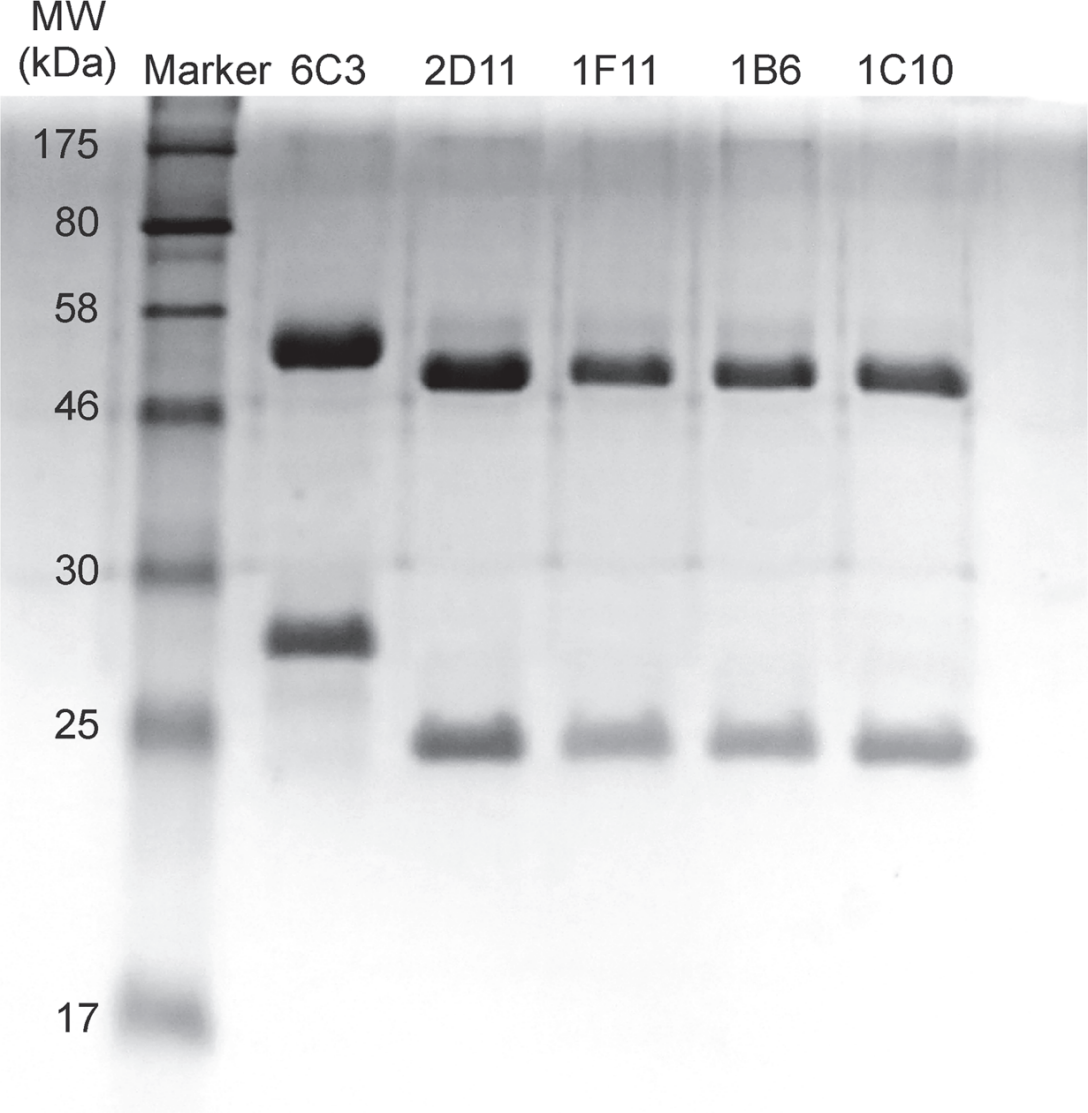
SDS-PAGE gel of purified Mabs. MW of the heavy chains are 52 kDa for 6C3; and 49 kDa for 2D11, 1F11 and 1C10. MW of the light chains are 28 kDa for 6C3; and 23 kDa for 2D11, 1F11 and 1C10.

The Mabs were selected, purified, and their reactivity with P7 investigated. Fig. 2 shows a 12% SDS-PAGE gel of the Mabs revealing the heavy and light chains of the purified IgG monoclonals. The heavy chain molecular weights are 52 KDa for 6C3; and 49 KDa for 2D11, 1B6, 1F11 and 1C10. The light chain molecular weights are 28 KDa for 6C3; and 23 KDa for 2D11, 1B6, 1F11 and 1C10. All five antibodies utilize the IgG1 subclass and the κ light chain as determined by ELISA. The variable region cDNA sequences derived from mRNA of the hybridomas were analyzed by IgBLAST and classification comparisons were made to the mouse database [29]. Sequence analysis reveals that Mabs 1C10, 1B6, 2D11, and 1F11 all utilize a V_κ_ derived from the same germline gene, V_κ_4-59*01 and a V_H_ gene derived from the V_H_5 family, either the V_H_5-9-3*01 or V_H_5-17*02 genes (Table 1). A germline analysis search demonstrates that 2D11 is similar to 1B6, and 1C10 is similar to 1F11 and therefore could be derived from the same myeloma fusion. In Table 1, the germline gene with the highest alignment is recorded. The restricted utilization of V_κ_ genes and V_H_ genes among 1F11, 1B6, 1C10, and 2D11 suggests that P7 elicits a limited antibody repertoire. We acknowledge that the mutational differences between the similar Mabs could be PCR artifacts, however, the lower binding affinities of 1B6 compared to 2D11, and 1F11 compared to 1C10, suggests that they are independent clones.

Mab 6C3 differs significantly from the other Mabs and utilizes the VK8–21*01 gene and the V2-9-1*01 heavy chain gene. Query of the IMGT gene database [30] for Mus musculus indicated that a significant number of antibodies utilizing the VK8-21*01 light chain variable region gene and the V2-9-1*01 gene have specificity for dsDNA. Antibody 6C3 most likely binds a strictly conformational epitope on P7 (see below) that could structurally mimic double stranded nucleic acid polymers thereby eliciting cross-reactivity with dsDNA or dsRNA. Antibody to another viral protein, EBNA-1 has previously been shown to cross-react with dsDNA [31,32]. However 6C3 displayed no cross-reactivity to either dsRNA or dsDNA in nucleic- acid based ELISA tests. It should be noted as well that a search of the database for antibodies utilizing the V_κ_4-59*01, or the V_H_5-9-2*01 or V_H_5-17*02 genes showed virtually no anti-DNA specificity.

### P7 binding characteristics of the monoclonal antibodies

**Fig 3:**
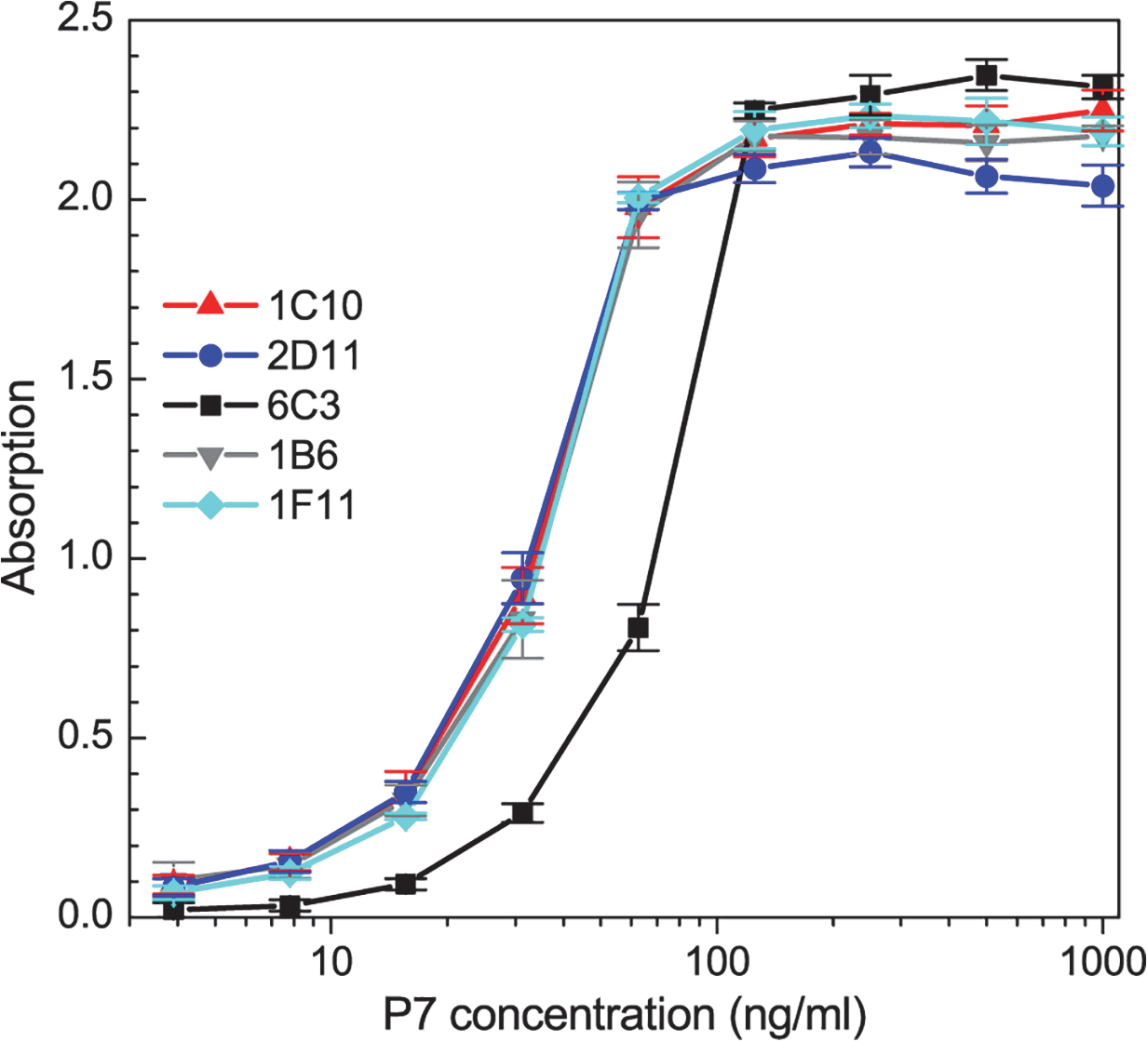
Affinity calibration curve for the P7 Mabs. Mabs concentrations were 1 μg/ml. The background absorption for each Mab was determined from the optical density of ELISA plate wells lacking the P7 antigen, and the background optical density was subtracted from the absorption data.

Fig. 3 shows the calibration curve for the binding affinity of each Mab to the native P7 antigen. The ELISA plates were coated with P7 native protein with concentrations ranging from 5 ng/ml to 1000 ng/ml (0.25 ng to 50 ng of P7 per ELISA well). The coating was performed at 4°C overnight to limit the rate of protein denaturation. We first performed preliminary assays to determine the optimal Mab concentration that produced a maximum optical response with the lowest background signal and found that concentration to be 1 μg/ml. Two distinct groups of antibodies, based on antigen affinity, were isolated which had the highest dynamic range for optical absorption. The groups were classified based upon the P7 antigen ELISA response with the following range of linear increase: 1) 20 ng/ml to 100 ng/ml for 6C3 antibody; and 2) 10 ng/ml to 50 ng/ml for the other four Mabs (Fig. 3).

**Fig 4:**
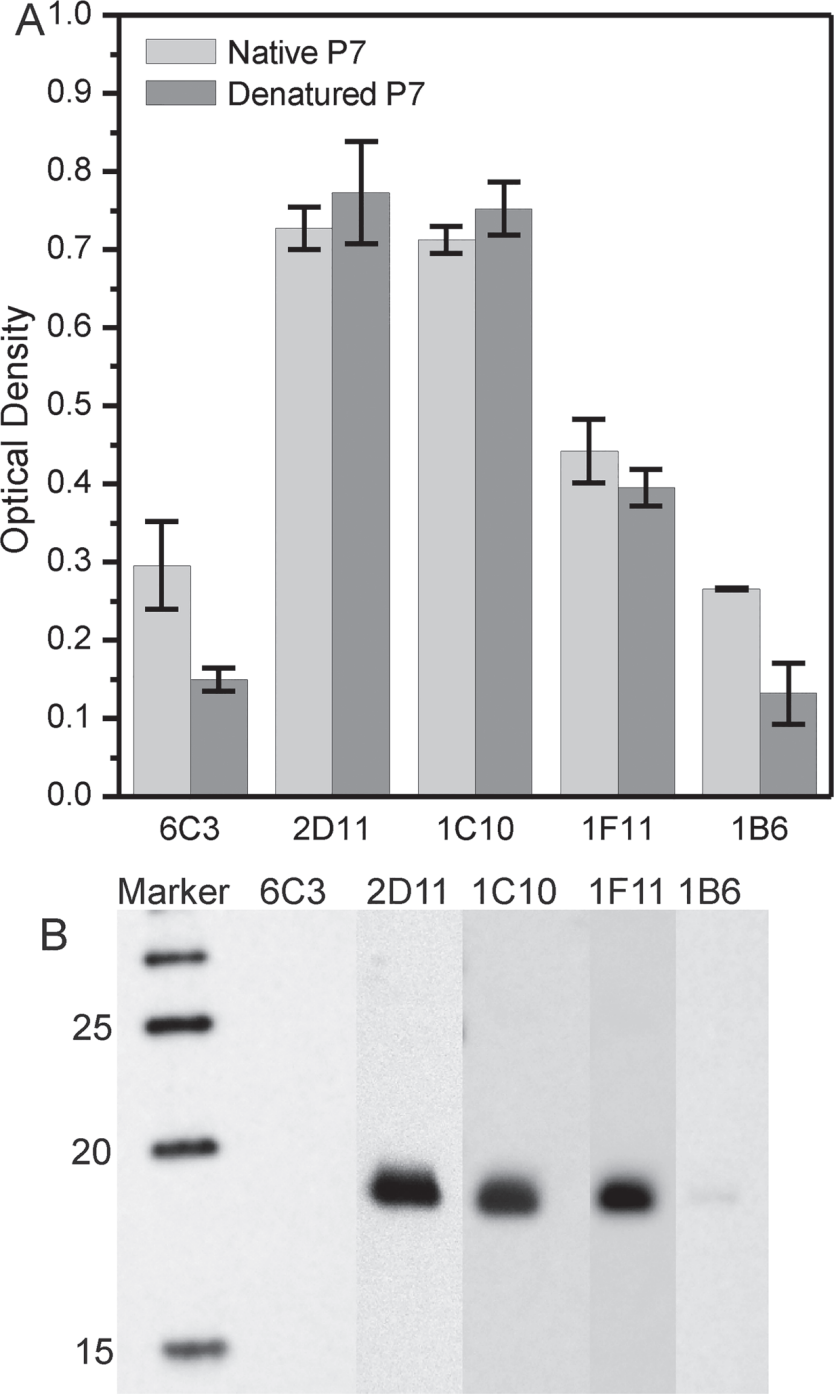
Reactivity of Mabs. A) Comparison of the reactivity of the Mabs 1C10, 6C3, 2D11 and 1F11 to the native and denatured forms of P7. P7 was coated at a concentration of 31.2 ng/ml (1.6 ng per ELISA plate well). Optical density is measured at 405 nm. B) Western Blot of P7 protein.

In order to categorize the epitopes as potentially linear or conformational for anti-P7 antibody, equal amounts of either native or denatured proteins were applied to ELISA plate wells. Each experimental group was performed in triplicate. Fig. 4 shows the optical density at 405 nm for an ELISA performed using both native and denatured P7 antigens. No significant difference in the reactivity of 1C10, 2D11, and 1F11 Mabs to native and denatured forms of P7 were observed. This suggests that these Mabs recognize linear epitopes of the P7 antigen. Significant differences in the reactivity of 6C3 and 1B6 between native and denatured P7 indicates that they likely recognizes a conformational epitope. Additional proof of this hypothesis is noted by the result of the Western Blot analysis in that the P7 band is visualized after 1C10, 2D11 and 1F11 labeling, but weak with 1B6, and almost no affinity with 6C3 labeling (Fig. 4B).

### Competition assays

**Fig 5:**
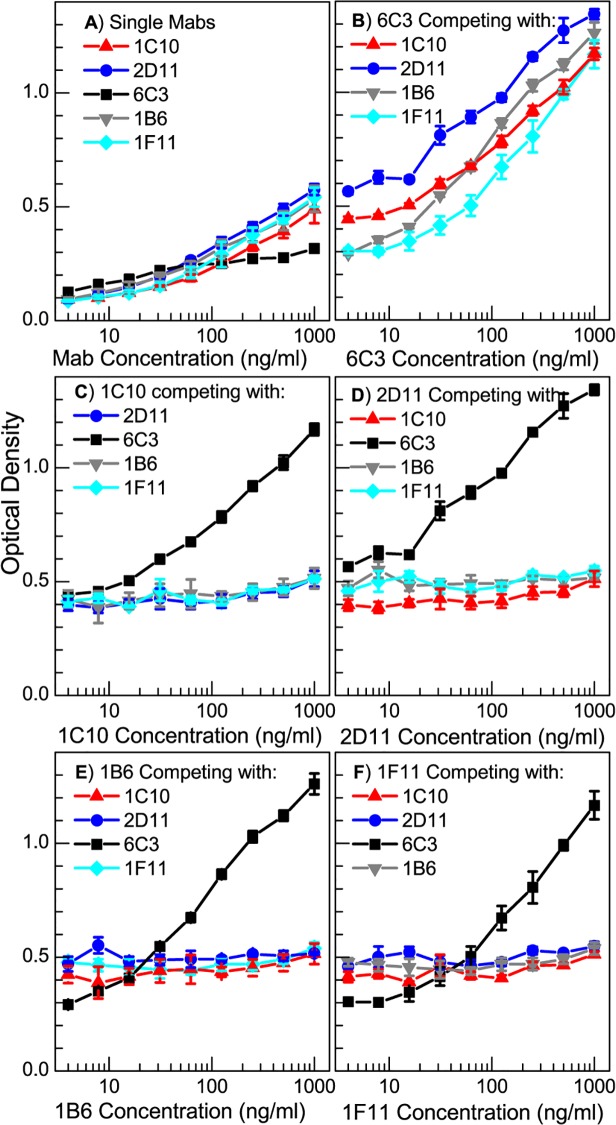
Competition between Mabs determined by ELISA. A) Single Mab (no competition); B) 6C3; C) 1C10; D) 2D11; and E) 1F11; in competition with the other three Mabs at concentrations of 1000 ng/ml. Optical Density is measured at 405 nm.

A competitive ELISA analysis was performed to determine if the Mabs competed for the same epitopes on P7. In the assays, 1.25 ng of native P7 was placed into each of four sets of wells of an ELISA plate. One μg/ml of each Mab was applied to each set of ELISA plate wells. Increasing concentrations of the other three competitor Mabs, were applied separately to each of the ELISA sets. Since all the Mabs are of the γ1 isotype, we cannot directly measure inhibition. Therefore if the saturating and the competitor antibodies are binding the same epitope, we would expect no change in net γ1 antibody binding to P7 even at very high concentrations of the competitor. However, if the saturating and the competitor antibodies are not competing for the same site and are binding different epitopes on P7, then one would expect to see an increase in γ1 antibody binding to P7 with increasing concentration of the competitor. As a control, increasing concentrations of individual Mabs were applied to sets of plates. The control absorption data is presented in Fig. 5A. Optical absorption for wells with saturating Mabs are shown in panels 5B through 5E (B: 6C3; C: 1C10; D: 2D11; E: 1F11). It is evident from Fig. 5B that the reactivity of 6C3 increases regardless of the competitor antibody, indicating a lack of competition between 6C3 and the other three Mabs. For each of the other three sets of wells containing saturating Mabs concentrations, absorption only increases for increased 6C3 concentration—consistent with the results presented in panel B. For none of the other combinations of Mabs does the absorption increase indicating that 1C10, 2D11 and 1F11 are all competing for the same sites on P7. Interestingly, the 6C3 reactivity is not additive with the other three antibodies as evidenced by the fact that optical densities of the mixtures of 6C3 with the other three Mabs is greater than the sum of optical densities of the individual reactions (Fig. 5A). This suggests there could be a synergistic cooperation between 6C3 and each of the other Mabs. It is conceivable that the antigen is structurally altered after binding with the initially added antibody better revealing the second epitope.

### Transcribing nucleocapsids can be captured in ELISA

The standard *in vitro* NC transcription reaction described by EmoriIba and Okada [28] was established to resemble the early-stage pattern of *in vivo* viral RNA synthesis in which greater proportion of l transcript is synthesized. The standard reaction is defined by its strict requirement for cations, pH, and l synthesis was found to be exceptionally sensitive to Mn^2+^ concentration. In addition the early stage of transcription could be associated with a limited or partially assembled P8 matrix. We suggest that the partial disassembly of the NC could occur in the standard reaction and if so the P7 would be in an exposed position. The monoclonal antibodies are ideal reagents for the capture of NC particles primed for transcription assuming the P7 is no longer shielded in the inner 3 fold axis of the procapsid [21] but has translocated to an open NC position post-RNA packaging.

**Fig 6:**
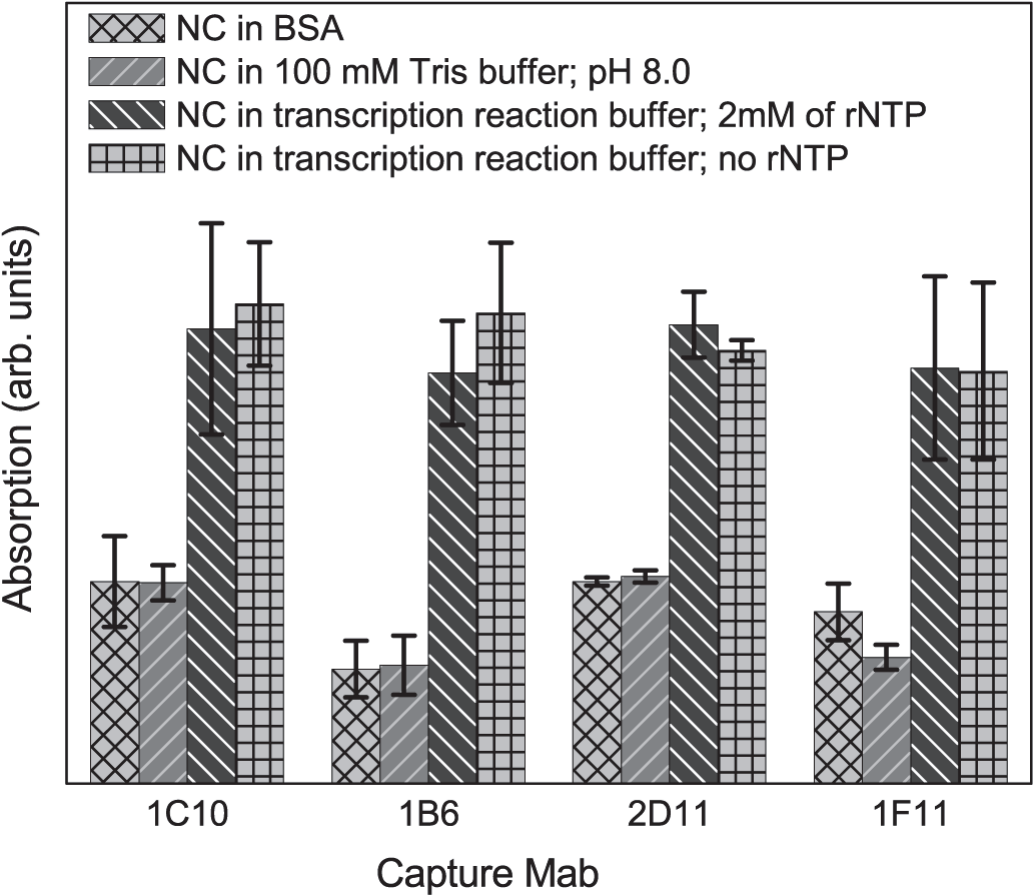
Sandwich ELISA showing that NC reveals P7 in transcription buffer with or without rNTP. Absorption is measured at 405 nm.

In order to determine if the Mabs were able to bind to the P7 protein *in situ*, we performed a sandwich—based ELISA. We chose as a capture antibodies 1C10, 2D11, and 1F11. Antibody 6C3—that recognizes a P7 epitope distinct from 1C10, 2D11, and 1F11- was biotinylated to serve as the detector. The ELISA plate wells were coated with capture antibody at a 5 ug/ml concentration. 50 ul of the NC early phase transcription reaction was applied to the first well and serial 2-fold dilutions in PBS of the reaction mixture were applied to the remaining wells in the ELISA plate row. Two control samples consisted of (1) NC in 100 mM Tris buffer (pH 7.5) and (2) NC in early phase transcription buffer but lacking rNTPs. Following the addition of the biotinylated 6C3 the plates were incubated with a 1:1000 streptavidin conjugated alkaline phosphatase. The substrate solution was added and the degree of NC capture was determined at 405 nm with a Titertek Multiscan ELISA reader. Fig. 6 shows the binding of the Mabs to ϕ6 NC samples after the transcription reaction and that only NCs that were placed into the early phase reaction were captured in the ELISA wells and detected with antibody 6C3. The capture was achieved in early phase buffer in the presence of absence of rNTPs indicating that the NC partial disassembly was nucleotide independent.

**Fig 7:**
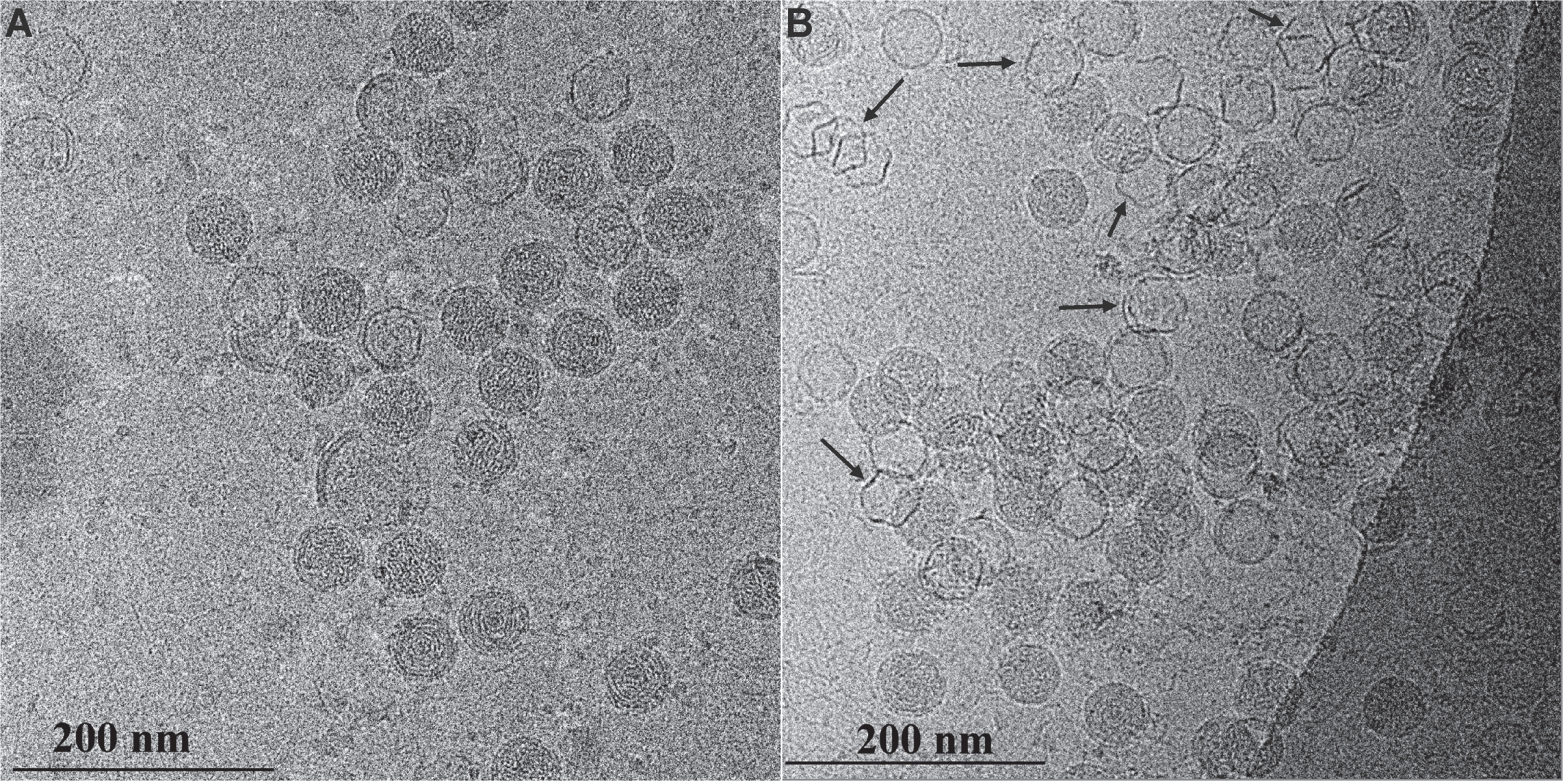
Cryo TEM images of transcribing ϕ6 NCs. A) Control sample (no rNTP in transcription reaction mixture); B) NC in 4 mM of rNTP in transcription reaction mixture. Arrows in (B) indicate some of the transcribing NCs (hexagonal configuration with a lower electron density).

We confirmed the partial disassembly of the NC using cryo-EM imaging of early phase transcription reaction mixtures. Cryo-EM projection images of the control (Fig. 7A) shows a large number of intact NCs which appear as 58 nm diameter particles with a high electron density due to the large contribution from the P8 matrix. A corresponding cryo-EM projection image of transcribing NCs (Fig. 7B) reveals that approximately 1/3 of the particles are in an intermediate state in which the P8 matrix is disassembled. NC transcription results in a virion appearing as an expanded hexagonal-shaped PC structure with a reduced internal electron density. Several of these transcribing particles are indicated by black arrows in Fig. 7B.

We note that the transcribing NC particles and controls (that are primed for transcription in the standard reaction conditions but lacking rNTPs) showed the increasing amount of available P7 in the ELISA reaction. The NCs maintained and tested in 100 mM Tris-HCl buffer (pH 8.0) did not have any evident immunolabeling.

The results of both the projection EM and sandwich assay analysis indicate that the P8 matrix is destabilized and likely completely or partially disassembled under *in vitro* transcribing conditions- either with or without 4mM rNTPs. The precise position of the transcribing viral RNA within the partially disassembled NC is not evident and its transit path through the portal remains to be determined. Notably the addition of Mabs 6C3 or 1C10 to NC transcribing reactions had no effect on synthesis of viral RNA transcripts.

We conclude the following from the assays: (1) monoclonal antibodies can be elicited from mice that recognize unique linear and conformational epitopes of protein P7; (2) Three of the four Mabs derived from distinct B-cell clones utilize identical V_κ_ and V_H_ genes of the same gene family demonstrating restricted gene utilization, while one, 6C3, which recognizes a conformational epitope, utilizes V_H_ and V_κ_ genes frequently associated with anti-DNA antibodies; and (3) monoclonal antibodies can be utilized alone or in combination as structural probes for the disassembly or assembly of the ϕ6 nucleocapsid during transcription in the standard early-stage reaction conditions. During NC transcription P7 is in an exposed position or in proximity to the NC surface which is accessible to antibodies.

## References

[1] Semancik JS, Vidaver AK, Van Etten JL. Characterization of segmented double-helical RNA from bacteriophage phi6. J Mol Biol 1973; 78: 617–625. 4357756 10.1016/0022-2836(73)90283-0

[2] Mindich L, Nemhauser I, Gottlieb P, Romantschuk M, Carton J, Frucht S, et al Nucleotide sequence of the large double-stranded RNA segment of bacteriophage phi 6: genes specifying the viral replicase and transcriptase. J Virol 1988; 62: 1180–1185, PMCID 253125. 3346944 10.1128/jvi.62.4.1180-1185.1988 PMC253125

[3] McGraw T, Mindich L, Frangione B. Nucleotide sequence of the small double-stranded RNA segment of bacteriophage ϕ6: novel mechanism of natural translational control. J Virol 1986; 58: 142–151, PMCID 252886. 3754015 10.1128/jvi.58.1.142-151.1986 PMC252886

[4] Nemecek D, Cheng N, Qiao J, Mindich L, Steven AC, Heymann JB. Stepwise Expansion of the Bacteriophage ϕ6 Procapsid: Possible Packaging Intermediates. J Mol Biol 2011; 414: 260–271, PMCID 3223026. 10.1016/j.jmb.2011.10.004 22019738 PMC3223026

[5] Butcher SJ, Dokland T, Ojala PM, Bamford DH, Fuller SD. Intermediates in the assembly pathway of the double-stranded RNA virus ϕ6. Embo J 1997; 16: 4477–4487. 9250692 10.1093/emboj/16.14.4477 PMC1170074

[6] Olkkonen VM, Bamford DH. The nucleocapsid of the lipid-containing double-stranded RNA bacteriophage phi 6 contains a protein skeleton consisting of a single polypeptide species. Journal of virology 1987; 61: 2362–2367, PMCID 255646. 3599179 10.1128/jvi.61.8.2362-2367.1987 PMC255646

[7] Olkkonen VM, Ojala PM, Bamford DH. Generation of infectious nucleocapsids by *in vitro* assembly of the shell protein on to the polymerase complex of the dsRNA bacteriophage phi 6. J Mol Biol 1991; 218: 569–581. 2016747 10.1016/0022-2836(91)90702-8

[8] Romantschuk M, Olkkonen VM, Bamford DH. The nucleocapsid of bacteriophage phi 6 penetrates the host cytoplasmic membrane. EMBO J 1988; 7: 1821–1829, PMCID 457174. 3169005 10.1002/j.1460-2075.1988.tb03014.x PMC457174

[9] Bamford DH, Mindich L. Electron microscopy of cells infected with nonsense mutants of bacteriophage ϕ6. Virology 1980; 107: 222–228. 7445427 10.1016/0042-6822(80)90287-1

[10] Huiskonen JT, de Haas F, Bubeck D, Bamford DH, Fuller SD, Butcher SJ. Structure of the bacteriophage ϕ6 nucleocapsid suggests a mechanism for sequential RNA packaging. Structure 2006; 14: 1039–1048. 16765897 10.1016/j.str.2006.03.018

[11] Mindich L. Packaging, replication and recombination of the segmented genome of bacteriophage ϕ6 and its relatives. Virus Res 2004; 101: 83–92. 15010219 10.1016/j.virusres.2003.12.008

[12] Qiao X, Qiao J, Mindich L. Stoichiometric packaging of the three genomic segments of double-stranded RNA bacteriophage phi6. Proc Natl Acad Sci USA 1997; 94: 4074–4079, PMCID 20570. 9108107 10.1073/pnas.94.8.4074 PMC20570

[13] Gottlieb P, Metzger S, Romantschuk M, Carton J, Strassman J, Bamford DH, et al Nucleotide sequence of the middle dsRNA segment of bacteriophage phi6: placement of the genes of membrane-associated proteins. Virology 1988; 163: 183–190. 3347997 10.1016/0042-6822(88)90245-0

[14] El Omari K, Sutton G, Ravantti JJ, Zhang H, Walter TS, Grimes JM, et al Plate Tectonics of Virus Shell Assembly and Reorganization in Phage Phi8, a Distant Relative of Mammalian Reoviruses. Structure 2013; 21: 1384–1395, PMCID 3737474. 10.1016/j.str.2013.06.017 23891291 PMC3737474

[15] El Omari K, Meier C, Kainov D, Sutton G, Grimes JM, Poranen MM, et al. Tracking in atomic detail the functional specializations in viral RecA helicases that occur during evolution. Nucleic Acids Res 2013, PMCID 3814363. 10.1093/nar/gkt713 PMC3814363 23939620

[16] Nemecek D, Qiao J, Mindich L, Steven AC, Heymann JB. Packaging accessory protein P7 and polymerase P2 have mutually occluding binding sites inside the bacteriophage ϕ6 procapsid. J Virol 2012; 86: 11616–11624, PMCID 3486324. 10.1128/JVI.01347-12 22896624 PMC3486324

[17] Juuti JT, Bamford DH. Protein P7 of phage ϕ6 RNA polymerase complex, acquiring of RNA packaging activity by *in vitro* assembly of the purified protein onto deficient particles. J Mol Biol 1997; 266: 891–900. 9086268 10.1006/jmbi.1996.0817

[18] Gottlieb P, Strassman J, Frucht A, Qiao XY, Mindich L. *In vitro* packaging of the bacteriophage phi 6 ssRNA genomic precursors. Virology 1991; 181: 589–594. 2014638 10.1016/0042-6822(91)90892-f

[19] Gottlieb P, Strassman J, Qiao XY, Frucht A, Mindich L. In vitro replication, packaging, and transcription of the segmented double-stranded RNA genome of bacteriophage ϕ6: studies with procapsids assembled from plasmid-encoded proteins. J Bacteriol 1990; 172: 5774–5782. 2211512 10.1128/jb.172.10.5774-5782.1990 PMC526894

[20] Sun X, Bamford DH, Poranen MM. Probing, by Self-Assembly, the Number of Potential Binding Sites for Minor Protein Subunits in the Procapsid of Double-Stranded RNA Bacteriophage ϕ6. J Virol 2012; 86, PMCID 3486473. 10.1128/JVI.01505-12 PMC3486473 22933292

[21] Katz G, Wei H, Alimova A, Katz A, Morgan DG, Gottlieb P. Protein P7 of the Cystovirus ϕ6 is Located at the Three-Fold Axis of the Unexpanded Procapsid. PloS One 2012; 7: e47489, PMCID 3471842. 10.1371/journal.pone.0047489 23077625 PMC3471842

[22] Poranen MM, Butcher SJ, Simonov VM, Laurinmaki P, Bamford DH. Roles of the minor capsid protein P7 in the assembly and replication of double-stranded RNA bacteriophage ϕ6. J Mol Biol 2008; 383: 529–538, PMCID 18793644. 10.1016/j.jmb.2008.08.082 18793644

[23] Eryilmaz E, Benach J, Su M, Seetharaman J, Dutta K, Wei H, et al Structure and dynamics of the P7 protein from the bacteriophage ϕ12. J Mol Biol 2008; 382: 402–422, PMCID 2744460. 10.1016/j.jmb.2008.07.006 18647606 PMC2744460

[24] Mindich L, Qiao X, Onodera S, Gottlieb P, Frilander M. RNA structural requirements for stability and minus-strand synthesis in the dsRNA bacteriophage ϕ6. Virology 1994; 202: 258–263. 8009837 10.1006/viro.1994.1341

[25] Mindich L, Qiao X, Qiao J, Onodera S, Romantschuk M, Hoogstraten D. Isolation of additional bacteriophages with genomes of segmented double-stranded RNA. J Bacteriol 1999; 181: 4505–4508. 10419946 10.1128/jb.181.15.4505-4508.1999 PMC103579

[26] de Haas F, Paatero AO, Mindich L, Bamford DH, Fuller SD. A symmetry mismatch at the site of RNA packaging in the polymerase complex of dsRNA bacteriophage ϕ6. J Mol Biol 1999; 294: 357–372. 10610764 10.1006/jmbi.1999.3260

[27] Tiller T, Busse CE, Wardemann H. Cloning and expression of murine Ig genes from single B cells. J Immunol Methods 2009; 350: 183–193. 10.1016/j.jim.2009.08.009 19716372

[28] Emori Y, Iba H, Okada Y. Transcriptional regulation of three double-stranded RNA segments of bacteriophage phi 6 in vitro. J Virol 1983; 46: 196–203, PMCID 255108. 6827650 10.1128/jvi.46.1.196-203.1983 PMC255108

[29] Ye J, Ma N, Madden TL, Ostell JM. IgBLAST: an immunoglobulin variable domain sequence analysis tool. Nucleic Acids Res 2013; 41: W34–40, PMCID 3692102. 10.1093/nar/gkt382 23671333 PMC3692102

[30] Lefranc MP, Giudicelli V, Ginestoux C, Bosc N, Folch G, Guiraudou D, et al IMGT-ONTOLOGY for immunogenetics and immunoinformatics. In Silico Biol 2004; 4: 17–29. 15089751

[31] Yadav P, Tran H, Ebegbe R, Gottlieb P, Wei H, Lewis RH, et al Antibodies elicited in response to EBNA-1 may cross-react with dsDNA. PLoS One 2011; 6: e14488, PMCID 3014975. 10.1371/journal.pone.0014488 21245919 PMC3014975

[32] Yadav P, Ebegbe R, Gottlieb P, Mumbey-Wafula A, Kaplan A, Spatz L. Antibodies elicited in response to EBNA-1 may cross-react with dsDNA. The Journal of Immunology 2010; 184: 93.29. 10.1371/journal.pone.0014488 PMC3014975 21245919

